# Reproductive endocrine characteristics and *in vitro* fertilization treatment of female patients with partial 17α-hydroxylase deficiency: Two pedigree investigations and a literature review

**DOI:** 10.3389/fendo.2022.970190

**Published:** 2022-09-14

**Authors:** Shutian Jiang, Yue Xu, Jie Qiao, Yao Wang, Yanping Kuang

**Affiliations:** ^1^ Department of Assisted Reproduction, Shanghai Ninth People’s Hospital, Shanghai Jiaotong University School of Medicine, Shanghai, China; ^2^ Department of Endocrinology, Shanghai Ninth People’s Hospital, Shanghai Jiaotong University School of Medicine, Shanghai, China

**Keywords:** 17-OHD, infertility, ppos, IVF-ET, reproductive endocrinology

## Abstract

**Background:**

17α-hydroxylase/17, 20-lyase deficiency (17-OHD) is caused by the mutations of the *CYP17A1* gene. The classical phenotype of 17-OHD includes hypertension, hypokalemia, and abnormal sexual development, with partial 17-OHD typically less severe than the complete deficiency. Infertility is always one of the main clinical manifestations of partial 17-OHD. However, to date, the pregnancy potentials of partial 17-OHD female patients have rarely been investigated, and few live-birth cases have been reported among them. Moreover, the reproductive endocrine characteristics of partial 17-OHD female patients have not been completely clarified and the treatment skills of *in vitro* fertilization and embryo transfer (IVF-ET) have not been well summarized yet.

**Methods:**

Two Chinese infertile female patients clinically diagnosed as partial 17-OHD were enrolled and their pedigree investigations were performed. Hormones were determined to depict the endocrine conditions of partial 17-OHD female patients. The adrenocorticotropic hormone (ACTH) stimulation test was performed to evaluate the functions of the adrenal cortex. Genotype analysis was conducted by next-generation sequencing (NGS) and Sanger sequencing was used to verify the results. IVF-ET was performed for the treatment of their infertility. Specifically, the progestin-primed ovarian stimulation (PPOS) protocol was chosen for the controlled ovarian hyperstimulation (COH) cycles, and the hormone replacement treatment (HRT) protocol was adopted for the endometrial preparation in frozen–thawed embryo transfer (FET) cycles.

**Results:**

Hormone assays revealed a reduced estradiol (E2) and testosterone (T) level, and an elevated progesterone (P4) level. The classic ACTH stimulating test evidenced a suboptimal response of cortisol to ACTH. Genotype analysis demonstrated that the proband1 carried two variants: c.1459_1467del (p.Asp487_Phe489del)^het^ and c.995T>C (p.lle332Thr)^het^. The proband2 was found to be a homozygote with the mutation of c.1358T>A (p.Phe453Ser)^hom^. The two female patients both succeeded in pregnancy and delivery of healthy babies through IVF-ET, with the usage of PPOS, HRT, and low-dose glucocorticoids.

**Conclusions:**

Partial 17-OHD female patients manifested menstrual cycle disorders and infertility clinically; displayed high P4 and low E2 and T; showed sparse pubic hair in physical examinations; and revealed multiple ovarian cysts in ultrasonic visualization. Moreover, the pregnancy potentials of infertile partial 17-OHD women seemed to increase with the adoption of IVF-ET. Considering the sustained elevated P4 level, PPOS is a feasible protocol for them in COH.

## Introduction

Congenital adrenal hyperplasia (CAH) encompasses a series of autosomal recessive disorders, characterized by enzymatic defects in the synthesis of cortisol (1). Among them, 17α-hydroxylase/17, 20-lyase deficiency (17-OHD) is considered the rarest one, with an estimated prevalence of 1 in 50,000–100,000, accounting for about 1% of CAH cases (2, 3).

This condition is caused by mutations within the cytochrome P450 family 17 subfamily A member 1 (*CYP17A1*) gene, located on chromosome 10q24-q25 (4). The encoded P450c17 includes the activities of both 17α-hydroxylase and 17, 20-lyase (5). The 17α-hydroxylase is a key enzyme required for the synthesis of cortisol and the 17, 20-lyase reaction is essential for the production of sex steroids (6). Therefore, *CYP17A1* mutations will lead to failure in cortisol and sexual hormone synthesis as well as high adrenocorticotropic hormone (ACTH) in plasma.

Clinically, the classic presentation of 17-OHD includes hypertension, hypokalemia, and abnormal sexual development (7). Disruption of sexual development affects men and women differently. In men, the deficiency causes feminization of external genitalia, and in women, it causes primary amenorrhea (gonadal dysplasia) (8). The severity of the features is variable (9). Two types of the condition are recognized: complete 17-OHD, which is more intensive, and partial 17-OHD, which is typically less severe but much rarer than the complete deficiency (10).

The diagnosis of 17-OHD is based on a comprehensive overview of clinical manifestation, serum hormone levels, and gene sequencing. However, the clinical and biochemical presentations of this disorder remain highly variable, and 10%–15% of patients are normotensive at diagnosis, which increases the difficulty of an accurate preliminary diagnosis (11). Adolescent girls usually seek help for delayed puberty (12). Women in the fertile stage usually seek help for primary infertility.

In fact, infertility is always a disturbing problem accompanying partial 17-OHD patients (13). Assisted reproductive techniques (ART) have been tried in women, but mostly without any success (13). The mechanism of infertility in 17-OHD female patients may be attributed to their abnormal hormones, with high progesterone and low estrogen levels inhibiting follicular and endometrial growth. ART can bypass a part of these defects, and some reports on successful live births of 17-OHD female patients through *in vitro* fertilization (IVF) support this theory (14–20).

Herein, we report two cases of very rare 46, XX partial 17-OHD who underwent IVF in our center using progesterone-primed ovarian stimulation (PPOS) and get a successful live birth. Meanwhile, we performed the pedigree analysis of these two probands to strengthen the association between genotypes and phenotypes in female patients with partial 17-OHD. Furthermore, combined with the literature review, we summarize the reproductive endocrine characteristics of 17-OHD women and the management skills in their IVF treatments.

## Materials and methods

### Basic information

The proband of the first family was a 29-year-old patient, who came to our assisted reproduction department after 3 years of primary infertility. The patient’s menarche was at the age of 16 and then she had oligomenorrhea for 13 years with cycles of 5 days/30–90 days. She suffered from recurrent bilateral ovarian cysts and had undergone two laparoscopic procedures to remove them. One was right ovarian cystectomy in October 2017, the other was left ovarian cystectomy in June 2018, and both of the postoperative pathological findings showed follicular cysts. The hysterosalpingography (HSG) in 2017 showed that her fallopian tube was obstructed on both sides, so when she received the second laparoscopic surgery in 2018, she underwent salpingoplasty and hydrotubation at the same time, after which her bilateral fallopian tube became partially obstructed. The parameters of her husband’s semen examination in our center were all within the normal range. She had received two cycles of ovarian stimulation treatment with clomiphene citrate in 2018 before coming to our center, during which the dominant follicle and ovulation were observed by transvaginal ultrasound (TVS), but she was unable to conceive.

The proband of the second family was a 31-year-old woman who had been infertile for 4 years. The patient had her menarche when she was 15 years old and her menstrual cycle was irregular, ranging from 25 days to 35 days. Each of her menstrual period lasted for 8 days while the menstrual flow was scanty. She underwent HSG in 2019 and the result showed that her bilateral fallopian tube was partially obstructed. Her husband’s semen test result displayed good density and motility.

The other family members in the pedigrees were referred to our department because they required clinical evaluation. Here, the patients underwent detailed examinations, including blood pressure measurements at rest, height and weight assessments, relevant blood tests (including G-banded karyotyping), and pelvic ultrasound and CT scanning.

### Determinations of hormones

Serum follicle-stimulating hormone (FSH), luteinizing hormone (LH), estradiol (E2), progesterone (P4), testosterone (T), prolactin, double hydrogen testosterone (DHT), and anti-mullerian hormone (AMH) levels were determined by chemiluminescent immunoassays. 17-hydroxyprogesterone (17-OHP), adrenocorticotropic hormone (ACTH), cortisol, and thyroid-stimulating hormone (TSH) were measured using radioimmunoassay at three different time points within 1 day (8 a.m., 4 p.m. and 12 a.m., respectively). The ACTH stimulation test was performed to evaluate the function of the adrenal cortex. In detail, Cosyntropin 25u (SPH No.1 Biochemical & Pharmaceutical, China) was injected intravenously, and blood samples were collected at 0, 30, and 60 min after the injections.

### Pedigree investigation and DNA sequencing

We conducted the pedigree surveys on the probands by interviewing the patients and their husbands and obtaining the patients’ family histories. The blood samples of these two families were collected. We intended to collect as many blood samples as possible from the family members and made the probands mobilize their entire families involved in the testing, but whether to accept the DNA sequencing in the end depended on the wishes of their family members. Genomic deoxyribonucleic acid (DNA) was isolated from the peripheral blood leukocytes of each family member. The entire coding region of the patients and their family members, including the exon–intron boundaries, was analyzed using next-generation sequencing (NGS). Variants that had a frequency <1% and affected amino acid coding or splice sites that were nonsynonymous changes were retained and further evaluated. Sanger sequencing was used to verify the results. The gene sequencing results were analyzed using Chromas software and compared with the corresponding sequences from the UCSC and NCBI databases to determine abnormalities in the *CYP17A1* gene.

### *In vitro* fertilization and embryo transfer treatment

**Figure 1:**
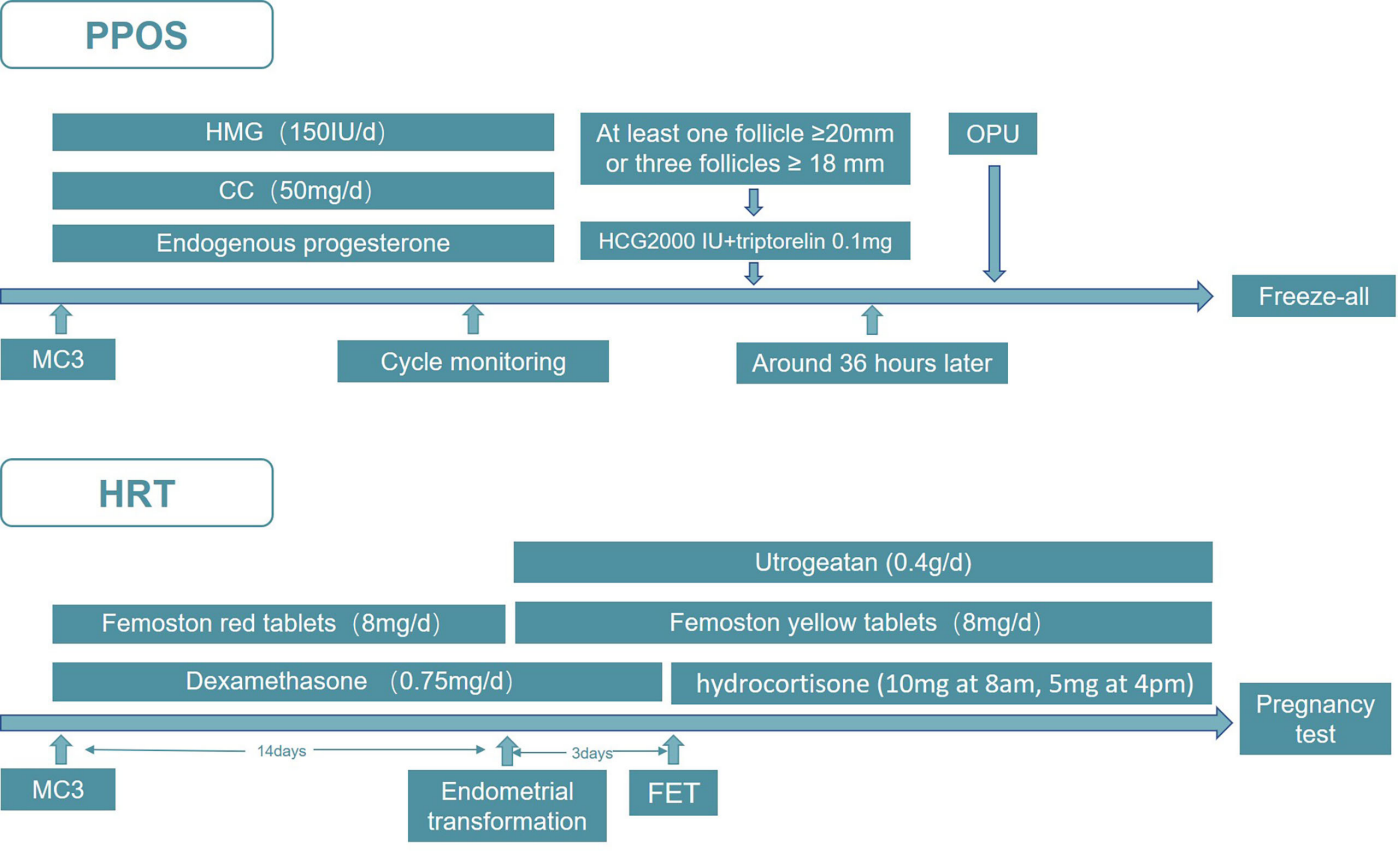
The PPOS and HRT protocol used in IVF-ET.

Since the two probands both visited our center for infertility, we started our treatment on them immediately after the clinical diagnosis was confirmed. In consideration of the unique reproductive characteristics of 17-OHD, together with their tubal factors, we decided to undertake *in vitro* fertilization and embryo transfer (IVF-ET) treatment. The PPOS protocol was adopted in the controlled ovarian hyperstimulation (COH) cycle in both of the patients. Specifically, as displayed in **Figure 1**, the patient was administered human menopausal gonadotropin (HMG, Anhui Fengyuan Pharmaceutical Co.) 150 IU/day initially. At the same time, patients were given Clomiphene citrate (Fertilan; Codal-Synto Ltd., France) 50 mg/day. When at least one dominant follicle reached 20 mm in diameter or three dominant follicles reached 18 mm in diameter, hCG 2000 IU (Lizhu Pharmaceutical Trading Co.) was used in combination with triptorelin 0.1 mg (Decapeptyl, Ferring Pharmaceuticals) to trigger ovulation. TVS-guided oocyte retrieval was performed around 36 h after the trigger. Day 3 embryos from IVF/ICSI treatment were graded according to the Cummins’ criteria, while the Gardner and Schoolcraft system was used to grade blastocysts. All of the viable embryos were frozen by vitrification, and hormone replacement treatment (HRT) was applied to endometrial preparation in the frozen–thawed embryo transfer (FET) cycle, in which dexamethasone was prescribed 0.75 mg/day before embryo transfer and hydrocortisone (10 mg at 8 a.m., 5 mg at 4 p.m.) was used after embryo transfer. From menstrual cycle day 3 (MC3) onwards, oral E2 (Femoston red tablets; Solvay Pharmaceuticals B.V.) 8 mg/day was given, with Femoston (yellow tablets; Solvay Pharmaceuticals B.V.) 8 mg/day and Utrogeatan 0.4 g/day starting the endometrial transformation. On the third day after endometrial transformation, embryos of good quality were transferred. The luteal-phase support continued until 12 weeks of the gestational week and the hydrocortisone was maintained throughout the pregnancy.

## Results

### Physical examination and auxiliary examinations

**Figure 2:**
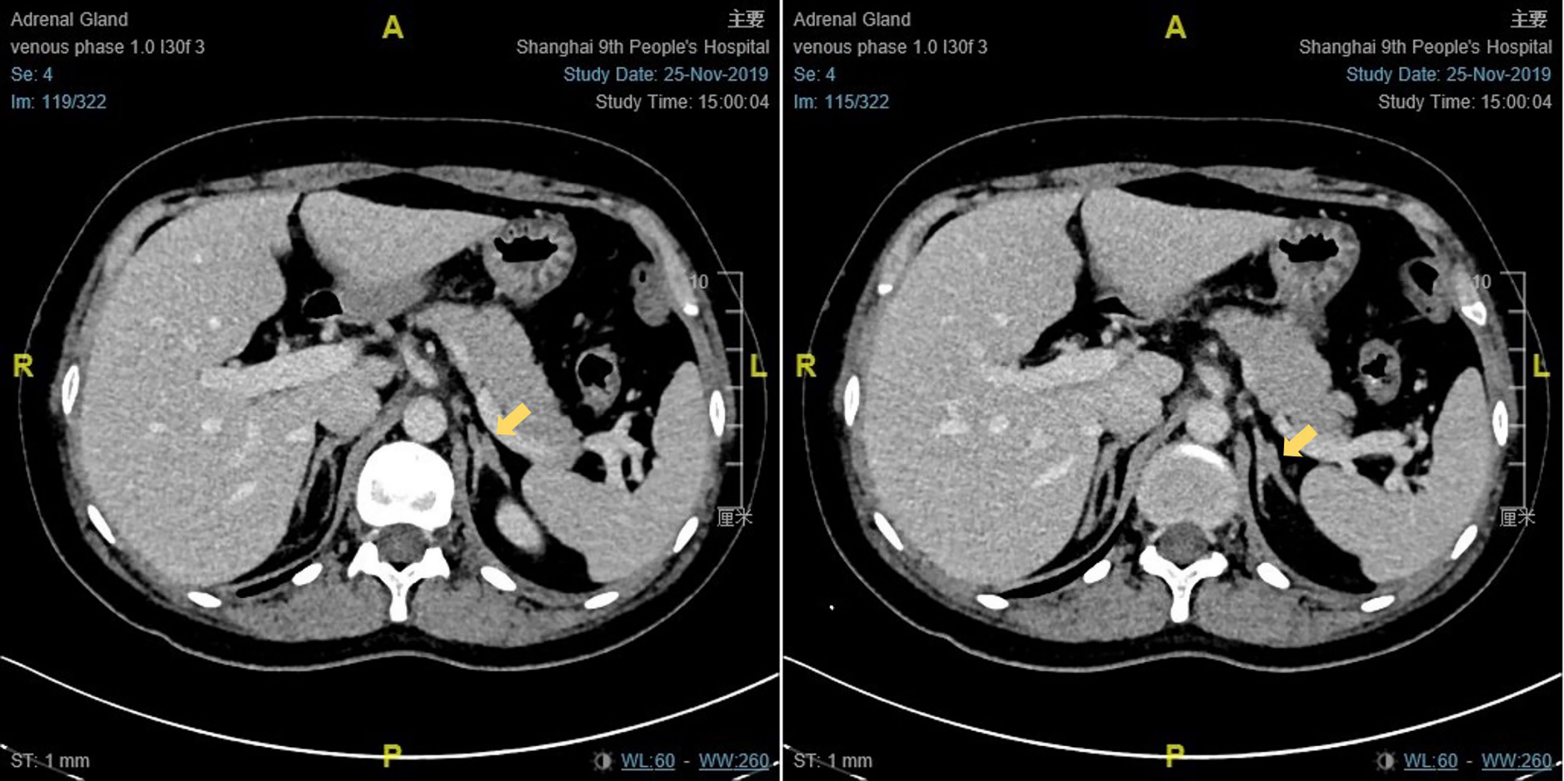
The enhanced CT scanning result of proband 1.

The results of physical examination and auxiliary examinations are detailed in **Table 1**. Proband 1 showed a height of 168 cm, a body weight of 67 kg, and a body mass index (BMI) of 19.1 kg/m^2^. Her blood pressure was 108/64 mmHg. Her breast development was in Tanner stage III and there was no pubic or axillary hair (Tanner stage I). Proband 2 presented with a height of 157 cm, a body weight of 55 kg, and a BMI of 22.31 kg/m^2^. Her blood pressure was 115/70 mmHg. Her Tanner stage assessments were Tanner stage III and Tanner stage I, based on the growth of breast and pubic hair, respectively. The external genitalia of the two patients were phenotypically female. Both of their pelvic ultrasounds revealed multiple ovarian cysts, together with a small uterus. Enhanced CT scanning of Proband 1 showed that the left adrenal junction was slightly thickened, about 10 mm wide, while the right adrenal gland presented with normal morphology and dimensions (**Figure 2**). The enhanced CT scanning of Proband 2 was normal. In addition, their chromosome karyotypes were both 46, XX.

**Table 1:** Physical examination and auxiliary examinations of the patients.

	Proband 1	Proband 2
Height (cm)	168	157
Body weight (kg)	67	55
BMI (kg/m^2^)	19.1	22.31
Blood pressure (mmHg)	108/64	115/70
Tanner stage assessment (breast)	III	III
Tanner stage assessment (pubic hair)	I	I
Pelvic ultrasonography	Multiple ovarian cysts and a small uterus	Multiple ovarian cysts and a small uterus
Adrenal enhanced CT	The left adrenal junction was slightly thickened	Normal
Karyotypes	46, XX	46, XX

BMI, body mass index.

### Hormonal measurements

Laboratory investigations disclosed the following common features (detailed values and reference ranges shown in **Table 2**): the serum FSH and LH were almost within the normal range, with the FSH level of proband 1 exceeding the normal upper limit; the E2 level was reduced, and the serum DHT and T were around the lower limit of the normal range, while the P4 was high (the assay was performed on menstrual cycle day 3 in follicular phase). The AMH was 1.43 ng/ml and 6.70 ng/ml, respectively. The patients also presented with normal blood potassium and sodium, as well as TSH. Furthermore, in the luteal phase, the patients took another three blood tests at 8 a.m., 4 p.m., and 0 a.m., respectively. The results showed increased ACTH, decreased 17-OHP and relatively normal level of cortisol, for all of the time points. The classic ACTH stimulating test evidenced a suboptimal response of cortisol to ACTH, while the dehydroepiandrosterone sulfate (DHEA-S) level maintained quite low (**Table 3**). Of note, if cortisol did not reach two times the basal value after ACTH stimulation, we considered it a blunt response and the patients may suffer from adrenal insufficiency (21).

**Table 2:** Laboratory hormone profiles of the patients.

	Proband 1	Proband 2	Reference range
FSH (mIU/ml)	10.44 (H)	6.59 (N)	Follicular phase:3.03-8.08
LH (mIU/ml)	3.28 (N)	4.52 (N)	Follicular phase:1.80-11.78
E2 (pg/ml)	10 (L)	27 (L)	Follicular phase:21-251
P4 (ng/ml)	4.20 (H)	2.10 (H)	Follicular phase:<0.3
T (ng/ml)	0.13 (N)	0.11 (N)	0.11-0.57
prolactin (ng/ml)	14.47 (N)	17.21 (N)	Non pregnant: 2.80-29.20
DHT (pg/ml)	15.60 (L)	14.90 (L)	15.6-142
AMH (ng/ml)	1.43 (N)	6.70 (N)	0.17-7.37
Blood potassium (mmol/l)	4.06 (N)	4.12 (N)	3.5-5.1
Blood sodium (mmol/l)	142 (N)	140 (N)	135-145
TSH (uU/ml)	3.18 (N)	2.68 (N)	0.35-4.94
ACTH 8 a.m. (pg/ml)	192 (H)	167 (H)	12-46
ACTH 4 p.m. (pg/ml)	38.9 (H)	43.2 (H)	6-23
ACTH 12 a.m. (pg/ml)	15.3 (H)	13.9 (H)	/
Cortisol 8 a.m. (ug/dl)	14.5 (N)	16.7 (N)	6.7-22.6
Cortisol 4 p.m. (ug/dl)	5.69 (N)	7.12 (N)	3.35-11.3
Cortisol 12 a.m. (ug/dl)	1.41 (N)	1.97 (N)	0-5.62

FSH, follicle-stimulating hormone; LH, luteinizing hormone; E2, estradiol; P4, progesterone; T, testosterone; DHT, double hydrogen testosterone; AMH, anti-mullerian hormone; TSH, thyroid-stimulating hormone; ACTH, adrenocorticotropic hormone.

**Table 3:** ACTH stimulating test results of the patients.

	Hormones	Before ATCH stimulating test	30min after ATCH stimulating test	60min after ATCH stimulating test
Proband 1	P4 (ng/ml)	3.20	3.50	3.60
	AD (ng/ml)	<0.30	<0.30	<0.30
	DHEA-S (ug/dl)	<15.00	<15.00	<15.00
	Cortisol (ug/dl)	1.45	15.2	15.5
	17-OHP (ug/dl)	0.57	0.59	0.62
Proband 2	P4 (ng/ml)	2.10	2.30	2.30
	AD (ng/ml)	<0.30	<0.30	<0.30
	DHEA-S (ug/dl)	<15.00	<15.00	<15.00
	Cortisol (ug/dl)	16.7	17.6	17.9
	17-OHP (ug/dl)	0.62	0.69	0.67

ACTH, adrenocorticotropic hormone; P4, progesterone; AD, androstenedione; DHEA-S, sulfated dehydroepiandrosterone; 17OHP, 17-hydroxyprogesterone.

### *CYP17A1* gene sequencing and pedigree analysis

We conducted pedigree surveys on the probands. Two kinds of pathogenic mutations were found in family 1 and another kind of mutation was detected in family 2 in the *CYP17A1* gene. The Sanger sequencing was performed to verify the mutations. The phenotypes and genotypes of the probands and their family members are detailed in **Table 4**.

**Table 4:** The phenotypes and genotypes of the patient’s family members.

Family 1	Phenotype	Genotype	Mutation type
Proband (III2)	Menstrual disorder, infertility, no pubic or axillary hair	c.1459_1467del/c.995T>C	Compound heterozygote
Father (II1)	Normal	c.1459_1467del/wt	Heterozygote
Mother (II2)	No pubic or axillary hair	Unknown	Unknown
Aunt (II3) (mother’s elder sister)	No pubic or axillary hair	c.995T>C/wt	Heterozygote
Aunt (II4) (mother’s younger sister)	No pubic or axillary hair	wt/wt	WT
Elder sister (III1)	Menstrual disorder, infertility, no pubic or axillary hair	c.1459_1467del/c.995T>C	Compound heterozygote
Elder female cousin (IV3)	Normal	wt/wt	WT
Family 2	Phenotype	Genotype	Mutation type
Proband (IV2)	Menstrual disorder, infertility, no pubic, or axillary hair	c.1358T>A/c.1358T>A	Homozygote
Father (III1)	Normal	c.1358T>A/wt	Heterozygote
Mother (III2)	Normal	c.1358T>A/wt	Heterozygote
Younger brother (IV3)	Normal	c.1358T>A/wt	Heterozygote
Grandmother (II2)	Normal	c.1358T>A/wt	Heterozygote
Maternal grandfather (II3)	Normal	c.1358T>A/wt	Heterozygote

**Figure 3:**
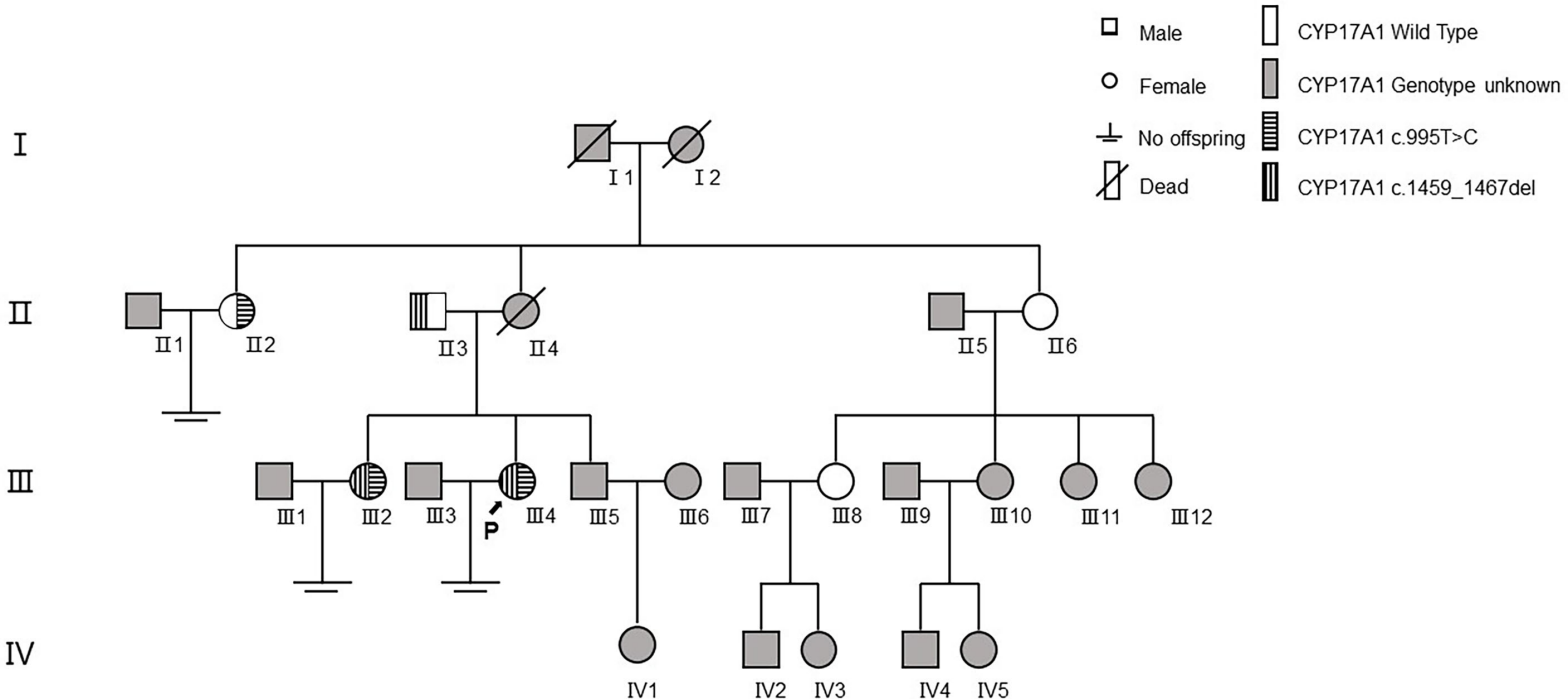
The family tree of proband 1.

In family 1 (**Figure 3**), the proband (III4) carried two variants: the missing mutation c.1459_1467del (p.Asp487_Phe489del)^het^ and the base substitution mutation c.995T>C (p.lle332Thr)^het^. Her elder sister (III2) harbored two identical genetic mutations, with similar clinical symptoms including infertility for 10 years, menstrual disorders (abnormal uterine bleeding history), recurrent multiple ovarian cysts, and an absence of pubic and axillary hair. These two variants were verified in their preceding generation. Their father (II3) carried c.1459_1467del^het^, without any special clinical manifestations. Although their mother’s genotype was unknown because she had passed away several years ago, we discovered her aunt (II2, mother’s elder sister) with the mutation of c.995T>C (p.lle332Thr)^het^. Her aunt also had no pubic or axillary hair, but she had regular menstrual periods. In the meantime, the patient’s other aunt (II6, mother’s younger sister) and her elder female cousin (III8) were proved to be wild type after gene sequencing. This aunt showed no pubic and axillary hair likewise, while her two grown daughters (III8&III10) were normal referring to body hair. Furthermore, the patient’s mother (II4) and maternal grandmother (I2) were absent of pubic or axillary hair, despite unknown genotypes.

**Figure 4:**
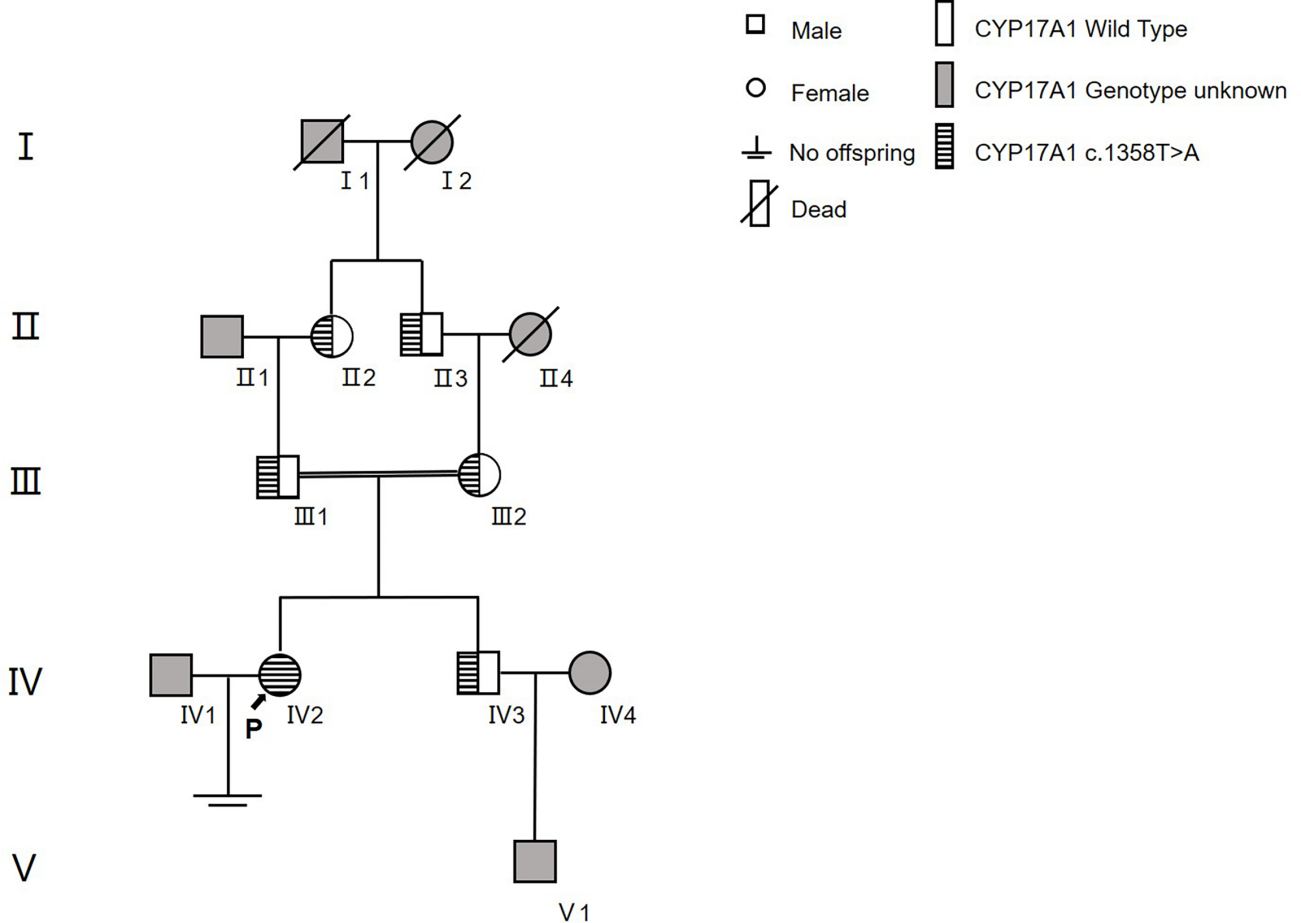
The family tree of proband 2.

In family 2 (**Figure 4**), the proband (IV2) was found to be a homozygote genetically with the mutation of c.1358T>A (p.Phe453Ser)^hom^. Her family had a history of consanguineous marriage, namely, her grandmother (II2) and maternal grandfather were brothers (II3) and sisters. Therefore, many members of her family all carried this disease-causing gene mutation (II2, II3, III1, III2, and IV3). However, because they were all heterozygotes [c.1358T>A (p.Phe453Ser)^het^], their clinical phenotypes were all normal. The younger brother of the proband was also a carrier of this mutation and already had a son.

### COH and FET cycle outcomes

We performed two controlled ovarian stimulation cycles (COH) and two FET cycles of proband 1. In the first COH cycle, the antral follicle count (AFC) was 3. A total of seven oocytes were achieved from 10 punctured follicles. Among them, six oocytes were in metaphase II (MII) and underwent intracytoplasmic sperm injection (ICSI) for fertilization. Five oocytes were fertilized and cleavaged, and three of them developed into high-quality embryos according to Cummins’s criteria (8CII*3). In the second COH cycle, the AFC was 4. Five follicles were punctured, three oocytes were derived (MII*3), three oocytes were fertilized through ICSI and cleavaged, and finally, three top-quality embryos were harvested (10CI, 8CI, and 7CII). In the FET cycle, two embryos of good quality were transferred (10CI and 7CII), resulting in an intrauterine singleton pregnancy. The patient chose to terminate the pregnancy due to ultrasonographic evidence of cleft lip and palate in the 23rd week of gestation, despite being properly informed about the risks of continuing the pregnancy versus ending it. In the case of the aborted fetus, whole-exon sequencing revealed heterozygous *CYP17A1* and *SLC12A3* mutations, which did not appear to be related to the phenotype of the fetus. The patient underwent the second FET cycle with another two top-quality embryos (8CII and 8CII). An intrauterine singleton pregnancy was successfully achieved again (**Supplementary Figure 1** shows the ultrasound images 4 weeks/6 weeks after embryo transfer). The pregnancy was uneventful without any pregnancy complications. Finally, a healthy male baby was delivered in January 2022, with a birth weight of 3,100 g and a birth length of 48 cm.

For proband 2, we performed one COH cycle and one FET cycle, which also resulted in a live birth. To be specific, in the COH cycle, 19 antral follicles were observed on MC3. After ovarian stimulation, 17 follicles were punctured and 10 oocytes were collected. Among them, seven oocytes were in the MII phase and received ICSI for insemination, which developed into four top-quality embryos for cryopreservation (8CII*2, 7CII, and 9CII). In the subsequent FET cycle, we chose two of the embryos for transfer (8CII*2). A smooth intrauterine singleton pregnancy was obtained, and finally, a healthy male baby was delivered in October 2021, with a birth weight of 3,300 g and a birth length of 50 cm.

## Discussion

Congenital adrenal hyperplasia is an autosomal recessive disorder due to a defect in any of the enzymes involved in the process of steroidogenesis. The most common cause of CAH is 21-hydroxylase deficiency (21-OHD) (22). Meanwhile, 17-OHD is rarely seen and accounts for only approximately 1% of all CAH cases. This is a complex disease that can be classified mainly into “complete” and “partial” type. Of the two types, partial deficiency is much rarer, with certain degrees of estrogenic and androgenic functions, which reduce the severity of the situation (23).

By means of pedigree survey and gene sequencing, we detected two mutations in family 1 and another mutation in family 2. Both proband 1 and her sister were compound heterozygotes with c.1459_1467del (p.Asp487_Phe489del)^het^ and t c.995T>C (p.lle332Thr)^het^ in family 1, and the proband 2 was a homozygote with c.1358T>A (p.Phe453Ser)^hom^ in family 2. To date, more than 100 mutations in exons and introns have been considered pathogenic or disease-causing mutations in 17α-OHD according to the ClinVar and HGMD databases.

For the part of clinical manifestations and physical examinations, the patients presented with oligomenorrhea, infertility, absence of pubic or axillary hair, and undeveloped breast, which were highly consistent with the characteristics mentioned in other cases (24, 25). Unlike complete 17-OHD women, the patient was not featured by primary amenorrhea or hypertension. Instead, they suffered from a series of problems involved in the reproductive systems, the difference of which was suggestive of a milder defect in partial 17-OHD.

The first heterozygous variant c.1459_1467del (p.Asp487_Phe489del) is located in Exon 8, which has been found homozygous or compound heterozygous in many patients leading to 17-OHD (21, 26–29) and co-segregated with disease in two Chinese families (30, 31). *In vitro* functional tests showed that the cells transfected with this variant had no 17α-hydroxylase activity or 17,20 lyase activity (26). This variant is included in HGMD and ClinVar as a “pathogenic variant” (ClinVar: 631622). According to the gnomAD database, the allele frequency of the mutation in the population database is currently less than 0.01% (9/274506). The allele frequency in the East Asian population is highest (0.05%), without homozygotes.

The second heterozygous mutation c.995T>C (p.Ile332Thr) is located in Exon 6. The mutation is in a compound hybrid form in a 17-OHD patient. *In vitro* functional studies have shown that the mutant protein still has a portion of 17α-hydroxylase activity (about 10%–25%) and a part of 17,20 lyase activity (about 10%) (32). This mutation is included in HGMD as a “pathogenic mutation” but not included in ClinVar. The gnomAD database shows that the allele frequency of this mutation in the population is currently less than 0.01% (5/282,842). The allele frequency is highest in the Latin population (<0.01%), and there is also no homozygote.

The third homozygous mutation c.1358T>A (p.Phe453Ser) is located in Exon 8 and has been reported to cause partial 17-OHD in a Chinese woman (28). It was revealed that the 17α-hydroxylase activity of this mutant protein was reduced to 29% of that of the wild type. This variant is included in HGMD and ClinVar as a “pathogenic variant”, but not included in the gnomAD database. The ClinVar database shows that the allele frequency of this mutation in the population is less than 0.01% (3/264,690).

Although pedigree investigation helped confirm the diagnosis through gene sequencing, still there was a confusing phenotype in family 1. The proband’s two aunts both presented with an absence of pubic or axillary hair, even though their genotypes were heterozygote (c.995T>C/wt) and wt/wt. The paradox was that the phenotype was mostly a character of 17-OHD, while the genotype was a symbol of normal 17α-hydroxylase activity and 17,20 lyase activity theoretically. This could be partially explained by family traits since hair thickness was individualized, and in this family, sparse hair might be idiopathic. Another possible explanation was that there was another gene mutation apart from *CYP17A1* related to lack of hair, such as *EPS8L3* accounting for hypotrichosis 5 (33).

Women diagnosed with 17-OHD have tried IVF-ET in the past few decades, but they have mostly failed (13). However, there are still some reports of successful live birth of this kind of patient through IVF-ET. Based on a comprehensive consultation of literature associated with IVF-ET treatments on 17-OHD women, we listed the following cases in chronological order (**Table 5**): a 33-year-old woman with 17-OHD and IVF-donated oocytes resulted in a live birth in 2003 (17); in the same year, an infertile 17-OHD woman achieved pregnancy and delivered three healthy babies with the help of IVF using her own oocytes (14); a successful live birth in a 26-year-old woman with IVF was documented in 2016 (20); two consecutive live births of a 24-year-old 17OHD woman followed (19); another successful live birth in a 26-year-old woman with IVF using was recorded in 2018 (18); Blumenfeld et al. described the achievement of the first successful pregnancy and delivery in a patient with 17,20-lyase deficiency (16); the first two Chinese cases of partial 17-OHD conceived and had a live birth through PPOS protocol (15). Notably, one of the cases in the last literature is just the proband 1 in our report (15). However, since the first report of this case did not include a complete pedigree survey and it was not centered on the patient’s reproductive endocrine characteristics, we paid more attention to her infertility and IVF-ET treatment in this report and described this case in detail again (the detailed treatment records are shown in **Supplementary Tables**). Moreover, we summarized the following reproductive-related commonalities in infertile partial 17-OHD women from our two cases together with these literature reviews.

**Table 5:** Previous literature of live births achieved by 17-OHD women undergoing IVF-ET.

Year	Author	Origin	Mutation	COH protocol	FET cycle	Diminish P4 production	Gestational weeks	Neonatal outcomes
2003	Ben-Nun I et al. (17)	Israel	NA	NA (donated oocytes)	HRT	Dexamethasone	25+4	A surviving male twin^a^ (weight 883 g)
2003	Levran D et al. (14)	Israel	NA	HMG	HRT	Long-acting GnRH-a + Dexamethasone	NA	Triplet live birth
2016	Bianchi PH et al. (20)	Brazil	p.W406R/P428L	Long GnRH-a protocol	HRT	Long-acting GnRH-a + Dexamethasone	30+4	Male (weight 1,945 g)
2018	Kitajima M et al. (19)	Japan	p.S54del/S54del	Short GnRH-a protocol	HRT	Dexamethasone	NA	Male (weight 3,980 g)
				Short GnRH-a protocol	HRT	Dexamethasone	39	Male (weight 3,972 g)
2018	Falhammar H et al. (18)	Sweden	NA (homozygote)	NA	NA	Prednisolone	37	Female (weight 3,290 g)
2021	Blumenfeld Z et al. (16)	Israel	p.E305G/E305G	long GnRH-a protocol	HRT	Long-acting GnRH-a + Prednisolone	41+2	Female (weight 3,650 g)
2022	Xu Y et al. (15)	China	p.I332T/D487_F489del	PPOS	HRT	Dexamethasone→Hydrocortisone	38	Male (weight NA)
		China	p.R496C/R496C	PPOS	HRT	Long-acting GnRH-a + Dexamethasone→prednisone	38+6	Female (weight NA)

a One of the twins dying within minutes of delivery.

17-OHD, 17α-hydroxylase/17, 20-lyase deficiency; IVF-ET, in vitro fertilization and embryo transfer; COH, controlled ovarian hyperstimulation; FET, frozen-thawed embryo transfer; P4, progesterone; NA, not applicable; HRT, hormone replacement treatment; GnRH-a, gonadotropin releasing hormone agonist; ACTH, adrenocorticotropic hormone.

The first distinctive reproductive endocrine feature shared by partial 17-OHD female patients was increased P4, which was usually clinically manifested as irregular menstruation and infertility. When coping with such a situation in IVF, we chose the PPOS protocol for ovarian stimulation in our cases. This protocol was originally proposed in 2015 (34) and had been proved to effectively prevent premature LH surges with progesterone supplementation in the early follicular phase, while achieving high-quality embryos and satisfactory pregnancy rates (35, 36). It had also been demonstrated that PPOS was a safe protocol without adverse effects on offspring (37). Therefore, this protocol had been widely adopted and included in the ESHRE Guideline in 2020 (38). Given the increased progesterone of partial 17-OHD women, PPOS seemed to be a suitable COH protocol, in which we happened to utilize endogenous progesterone to suppress LH surges instead of exogenous additions. Furthermore, it is unnecessary to strictly suppress progesterone and keep it within the normal range throughout the COH process. Moreover, compared to the GnRH-agonist protocol applied in the previous cases, PPOS presents the advantages of a simpler procedure, shorter stimulation duration, as well as a less financial investment.

Meanwhile, to address the impact of increased progesterone on endometrial preparation during the embryo transfer cycles, a freeze-all strategy was taken. All viable embryos were frozen by vitrification and FET was performed using the HRT protocol subsequently, which is in agreement with the regimen applied in the former cases. Generally, corticosteroid was prescribed to keep progesterone at relatively low levels before endometrium transformation. In our case, we used dexamethasone 0.75 mg/day from the last menstrual cycle to the day before embryo transfer, and after embryo transfer, hydrocortisone (10 mg at 8 a.m., 5 mg at 4 p.m.) was used ever since through the gestation. However, in the previous two cases in which the patient had a live birth, dexamethasone was used throughout the pregnancy without change. It should be noticed that, unlike hydrocortisone and prednisolone, dexamethasone crosses the placental barrier to the fetus without inactivation (39). In our preceding study on 21-OHD patients undergoing IVF, we recommend stopping the administration of dexamethasone after embryo transfer to avoid possible adverse influences on the fetus (40).

The second noticeable reproductive endocrine characteristic of partial 17-OHD women was decreased E2. In the previous cases, very low estradiol had also been noticed (19, 20). Centered on this abnormal sexual hormone, the following problems may arise for the reproductive system and COH process.

First of all, weakened negative feedback of estrogen to the pituitary gland may result in slightly increased FSH and LH levels, which make the patients more prone to develop ovarian cysts. However, considering the high P4 levels, the feedback weakening and the increases in gonadotropins might not be so obvious. What we could observe is usually a slightly elevated FSH level relative to the ovarian reserve. In our report, the cases all had relatively high FSH levels as well as a history of ovarian cyst operation before they came to our hospital. Since ovarian surgery might cause injury to the ovarian function, the ovarian reserve of our patient (proband 1) was relatively low with an AMH value of 1.43 ng/ml and an AFC of 3. However, the case reported in 2016 documented an AFC of 32, despite the limited number of dominant follicles (20); the women recorded in 2018 presented with hyper-response with 21 oocytes retrieved (19), although both of the cases had a lack of ovarian surgery histories. It reminded clinical physicians that when confronted with ovarian cysts in partial 17-OHD women, which seems to be a sticky condition requiring sophisticated and accurate treatment, they need to take a comprehensive diagnosis, strictly obey operation indications, and prudently manipulate, to avoid further damage to fertility. Next, the extremely low estradiol level of partial 17-OHD women made it difficult for physicians to evaluate the follicle development situations as well as the appropriate timing for triggering in the COH process. Therefore, we could only use follicular diameter rather than estradiol level as the indicator to make adjustments to the drug dosage in COH and determine when to perform oocyte retrieval. Moreover, it is perceived by some that complete estradiol deficiency causes follicular developmental arrest (13). Therefore, a small dose of estradiol was applied to COH in one of the previous cases. However, evidence also shows that favorable oocyte maturation and embryo development can occur in a low estradiol environment (41). Given that all of the cases produced high-quality embryos resulting in a live birth, even if without the external supplementation of estrogen, it appeared that reduced estradiol had no adverse effect on the development and maturation of follicles in partial 17OHD women, or on the embryo developmental potentials.

The third silent trait of reproductive endocrinology in partial 17-OHD women was decreased T, which often presented with sparse hair clinically. Since it is in the female population, low testosterone had very little effect on reproductive system development and fertility. Nevertheless, this feature can still inspire clinicians that if no pubic hair or sparse pubic hair is noticed during the gynecological examination, it is necessary to pay attention to the growth of other body hair and not miss the diagnosis of 17-OHD.

An intriguing phenomenon is that the fallopian tubes of the two patients were all partially obstructed. Meanwhile, the infertile woman in family 1 (III2) also had infertility, with her fallopian tubes partially obstructed too, although her husband had oligospermia. However, since previous reports did not mention the results of tubal patency, we could not reach a conclusion that fallopian tube obstruction was a common finding based on the two cases. Instead, we thought of it as a chance result.

Moreover, the laboratory assays also demonstrated reduced 17-OHP and elevated ACTH, with the ACTH stimulating test revealing an inadequate response, explaining the thickened adrenal on CT scanning. These observations were in accordance with those in previous literature (10, 24, 25, 42). However, the potassium concentration and sodium concentration were normal in our results, while most of the cases documented declining potassium in the blood (23–25, 42).

Furthermore, it should not be neglected that endometrial cavity fluid (ECF) appeared repeatedly in our patients in both COH cycles and FET cycles. This phenomenon had not been referred to in any previous literature. As it was commonly recognized that ECF has a negative influence on implantation and pregnancy, it was a troublesome problem that need to be solved. Fortunately, the ECF disappeared spontaneously the day before embryo transfer in the return visit in our cases. Otherwise, we might perform aspiration of uterine effusion once we confirmed the existence of ECF. The causative reasons for ECF were not completely understood and were speculative to be a tubal factor, polycystic ovarian disease, subclinical uterine infections, and so on (43, 44). For our patients, the formation causes of ECF also remained unknown, possibly relevant to 17-OHD itself due to the consistency of the manifestations of our cases.

## Conclusion

In conclusion, we are among the first to report the live births of infertile women diagnosed with partial 17-OHD, who underwent IVF-ET using the PPOS protocol. Meanwhile, we conducted two pedigree investigations to further clarify the genotypes and phenotypes of these patients. Considering the sustained elevated P4 level, PPOS is a feasible regimen for 17-OHD patients in COH, while it is also convenient, efficient, and economic. Furthermore, we made a systematic review of the cases of infertile 17-OHD women who achieved pregnancy and live birth through IVF-ET. Taken together, we depicted and concluded the reproductive characteristics of partial 17-OHD women clinically and biochemically. In summary, clinical manifestations include menstrual cycle disorders and infertility; endocrine hormone examination displays high progesterone, low estrogen, and low testosterone; physical examination shows sparse pubic hair; and ultrasound examination reveals multiple ovarian cysts. Based on these features, it is vital to make an early but accurate diagnosis in women of this disorder to help them obtain reproductive success.

## Data availability statement

The data presented in the study are deposited in the Genebank repository, accession number ON815636-ON815639.

## Author contributions

YK, YW, and JQ contributed to the conception and design of the study. SJ and YX analyzed the data and drafted the manuscript. All authors participated critical discussion and reviewed the manuscript. All authors contributed to the article and approved the submitted version.

## Funding

This study was funded by the Clinical Research Program of 9th People’s Hospital, Shanghai Jiao Tong University School of Medicine (JYLJ2019015), Shanghai Jiao University Scientific and Technological Innovation Funds (17JCYA01), and National Key Research and Development Program of China (2018YFC1003000).

## Conflict of interest

The authors declare that the research was conducted in the absence of any commercial or financial relationships that could be construed as a potential conflict of interest.

## Publisher’s note

All claims expressed in this article are solely those of the authors and do not necessarily represent those of their affiliated organizations, or those of the publisher, the editors and the reviewers. Any product that may be evaluated in this article, or claim that may be made by its manufacturer, is not guaranteed or endorsed by the publisher.
