# Supplementary figures and images for: Reproductive endocrine characteristics and in vitro fertilization treatment of female patients with partial 17α-hydroxylase deficiency: Two pedigree investigations and a literature review
Source: Front Endocrinol (Lausanne). 2022 Sep 14;13:970190. doi: 10.3389/fendo.2022.970190 (PMC9516945; doi:10.3389/fendo.2022.970190)

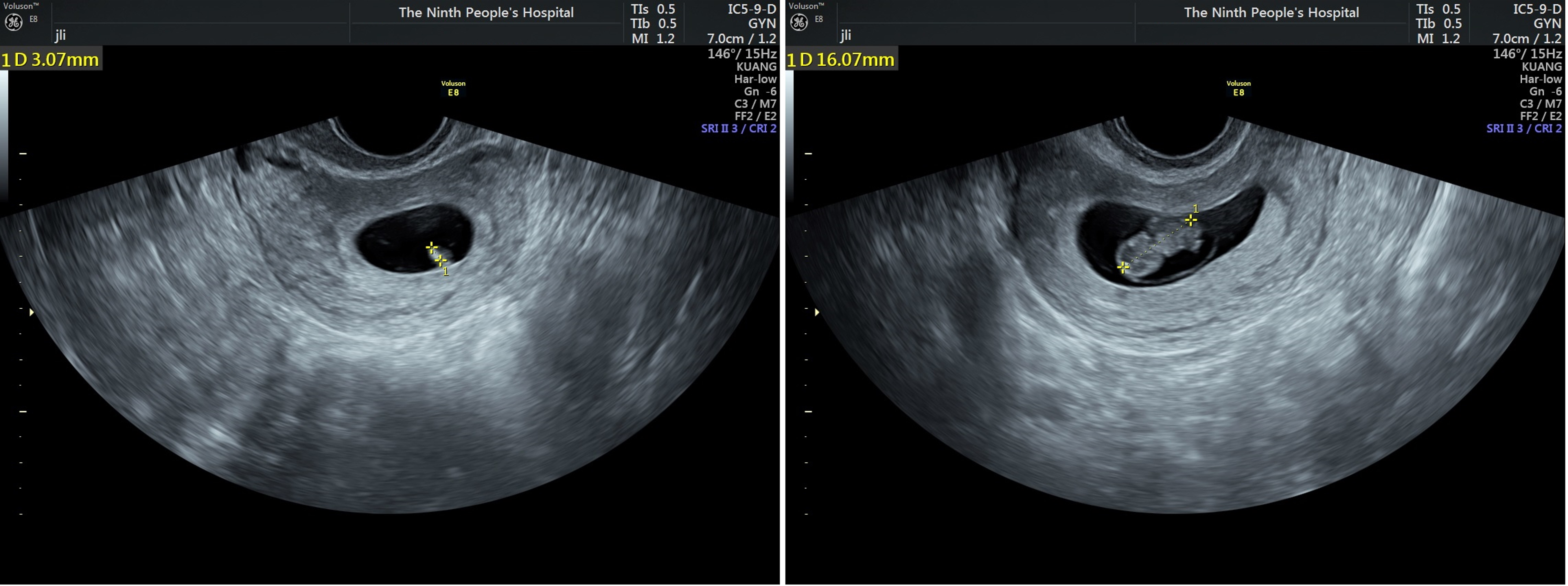

Supplement: Supplementary file 1 [file Image_1.jpeg]
